# Imperforate Anus with Jejunal Atresia Complicated by Intestinal Volvulus: A Case Report

**DOI:** 10.21699/jns.v5i4.458

**Published:** 2016-10-10

**Authors:** Hae Soo Joung, Alexandra Leon Guerrero, Sandra Tomita, Keith A. Kuenzler

**Affiliations:** Division of Pediatric Surgery, Department of Surgery, NYU Langone Medical Center, NYU School of Medicine, New York, NY

**Keywords:** Anorectal malformations, Imperforate anus, Jejunal atresia, Segmental volvulus

## Abstract

Anorectal malformations (ARMs) commonly co-occur with other congenital anomalies, particularly VACTERL (vertebral, anorectal, cardiac, tracheal, esophageal, renal, limb, and duodenal) associations. However, this collection of associations is not comprehensive, and other concurrent anomalies may exist that can be missed during the standard work-up of patients with ARMs. We present a rare case of a neonate with a low ARM with concurrent jejuno-ileal atresia that was diagnosed after the correction of the ARM when the patient developed segmental volvulus. This case illustrates the importance of having a high index of suspicion when deviation from a classic presentation occurs.

## INTRODUCTION

The incidence of anorectal malformations is approximately 1 in 5000 live births. [1] The incidence of jejunoileal atresia is around 1 in 5000 to 1 in 14,000 live births. [2] Approximately 50% of neonates with ARMs have one or more abnormality affecting another organ system; however, it is exceedingly rare for an anorectal malformation and intestinal atresia distal to the duodenum to occur in the same patient. [3,4] We report a case where an anorectal malformation with a median raphe perineal fistula was found to have a concurrent jejuno-ileal atresia, which presented as segmental volvulus. 

## CASE REPORT

A term male infant was born via normal spontaneous vaginal delivery. During initial examination, the baby was found to have an imperforate anus with the classic findings of a low anorectal malformation including a "bucket-handle" malformation and a median raphe fistula. The patient was hemodynamically stable. The abdomen was soft, non-tender, and non-distended. The patient urinated normally. No meconium was passed within the first 24 hours after delivery, and no meconium could be seen bulging through the perineum or median raphe fistula. Initial chest and abdominal radiographs demonstrated gas within the stomach and proximal small bowel, but no gas in the lower abdomen, colon, or rectum. A nasogastric tube was passed into the stomach and position confirmed on an abdominal radiograph. An echocardiogram revealed no congenital cardiac anomalies. 

The patient underwent a posterior sagittal anorectoplasty after 24 hours of examination. The perineal fistula was confirmed and the procedure was uncomplicated. However, no meconium was encountered within the rectum during or after the repair. Eight hours post-operatively, the patient was noted to have a 25-point hematocrit drop to 14.6 from 39.7. He was immediately resuscitated with blood and crystalloid, remained hemodynamically stable, but was re-intubated for airway protection. Examination revealed a moderately distended abdomen, while the surgical site was intact without signs of hemorrhage. A nasogastric tube was placed with return of bilious output. AP and cross-table lateral films of the abdomen were obtained with no evidence of free air, and still no gas seen within the lower abdomen. Abdominal ultrasound was performed demonstrating a large space-occupying tubular structure in the pelvis spanning 4.95 cm in diameter, a normal caliber rectum, and mild hydroureter and hydronephrosis. 

The patient was taken for an exploratory laparotomy, which revealed a type IIIa jejuno-ileal atresia with a severely dilated proximal end, and an associated intestinal volvulus about a narrowed segment of mesentery adjacent to the atresia. There was no blood within the peritoneal cavity, so the patient was presumed to have hemorrhaged into the proximal blind-ending loop of small bowel (Fig.1). The bowel was detorted and no malrotation was found. The necrotic segment of bowel, including the atretic segment, was resected, an end jejunostomy was created, and the terminal ileum was closed and marked with a non-absorbable suture for use in future reconstruction. 

The patient was transferred to the neonatal intensive care unit post-operatively where he had an uneventful recovery. Within 2 weeks he was tolerating full oral feeds, having good ostomy function, and weaned from parenteral nutrition. His anoplasty healed well and he was started on dilations. He underwent an ostomy reversal with primary jejunoileal anastomosis 6 weeks after his jejunostomy creation. Today, the patient continues to tolerate full oral feeds and is having normal growth. 

**Figure:**
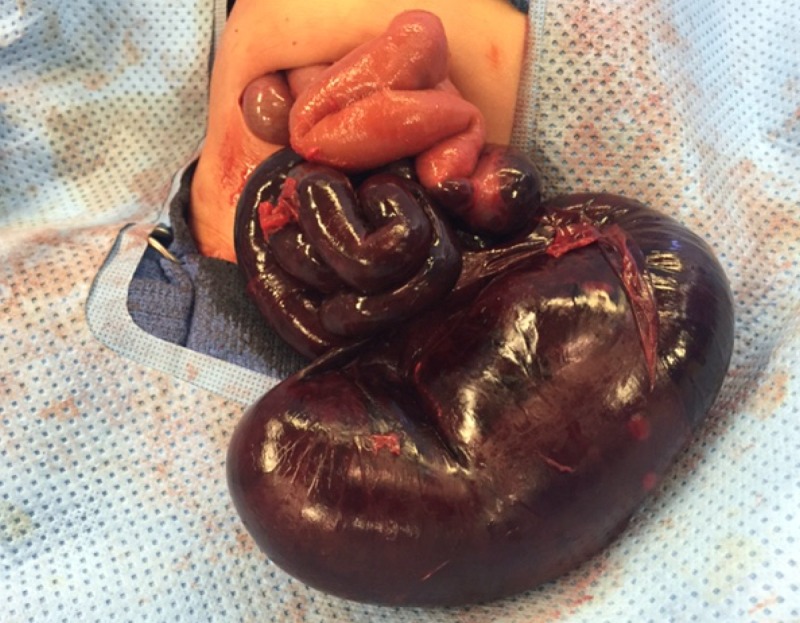
Figure 1: Jejunal atresia with dilated, necrotic proximal end.

## DISCUSSION

There are a total of 8 previously reported cases of ARMs associated with jejunoileal atresia. [3, 5] To our knowledge, this is the first report of a patient with a coexisting anorectal malformation and jejunal atresia complicated by volvulus. The reported incidence of jejunal/ileal atresia presenting with volvulus ranges from 25% to 27%. [2, 6] Alimentary tract atresias involving both the midgut and hindgut in the same patient are rare and it is presumed that the embryogenesis of each is different. Abnormalities of the rectum and anus are thought to be due to arrest of the caudal descent of the urorectal septum to the cloacal membrane. The resulting malformations range from isolated imperforate anus to persistent cloaca and the spectrum of anomalies is thought to result from the timing of embryological developmental arrest. [7]

Intestinal atresias are often suspected in the antenatal period, as they can present with bowel dilation and polyhydramnios in the 3rd trimester of pregnancy. However, this is not highly sensitive or specific because other conditions, including imperforate anus, can present with similar prenatal ultrasound features. [6] In our case, there were no antenatal abnormalities that suggested a possible alimentary tract obstruction. Postnatally, intestinal atresia presents with bilious emesis, and timing of the presentation typically correlates with the level of the atresia. Whereas duodenal atresias present within the first 24h after birth, distal atresias may present after 48h. [8] In our patient, his anorectal malformation was surgically addressed within 48 hours after delivery, which can be before the time one may expect the presentation of a distal intestinal atresia.

This case describes a rare co-occurrence of ARMs and jejunoileal atresia, and illustrates the importance of maintaining a high index of suspicion when deviations from a classic presentation of either entity occur. In hindsight, the lack of meconium noted within the rectum and the absence of gas in the colon on abdominal radiograph after 24 hours of life was indicative of a proximal obstruction.

## Footnotes

**Source of Support:** Nil

**Conflict of Interest:** Nil
